# Association between sleep–wake habits and use of health care services of middle-aged and elderly adults in China

**DOI:** 10.18632/aging.102860

**Published:** 2020-02-24

**Authors:** Beizhu Ye, Yimei Zhu, Xiaoyu Wang, Sheng Wei, Yuan Liang

**Affiliations:** 1 School of Public Health, Tongji Medical College, Huazhong University of Science and Technology, Wuhan, Hubei 430030, China; 2 School of Media, Communication and Sociology, University of Leicester, Leicester LE1 7JA, UK

**Keywords:** circadian rhythms, sleep habits, sleep-wake cycles, health care services, physician visits

## Abstract

Objective: To examine the relationship between sleep-wake habits and the use of health care services.

Results: The proportions of the participants who were “early to bed” and “late to bed” were 48.7% and 51.3%, respectively. In the full sample, compared with those who were early to bed and early to rise, participants who went to bed late were more likely to report physician visits (late to bed and early to rise: OR = 1.13, 95% CI: 1.08–1.19, late to bed and late to rise: OR = 1.27, 95% CI: 1.18–1.38, respectively). We found no significant association between sleep-wake habits and the number of hospitalization.

Conclusions: Those middle-aged and elderly people who stayed up late and got up late are more likely to visit the doctors than those who went to bed early and got up early.

Methods: We obtained data from a cohort study of retired employees in China, and 36,601 (95.59%) involved in the present study. The participants were allocated into 4 sleep-wake habits groups: Early-bed/Early-rise, Early-bed/Late-rise, Late-bed/Early-rise, and Late-bed/Late-rise. We explored the association between sleep-wake habits with the number of physician visits and hospitalizations.

## INTRODUCTION

Aphorisms such as “Early to bed and early to rise makes a man healthy, wealthy, and wise” and “An apple a day keeps the doctor away,” are considered the crystallization of practical experience and wisdom of broad masses of people. Some aphorisms, such as “Early to bed and early to rise…” have come to symbolize health and healthy habits, serving to guide people’s lifestyles for centuries—or even thousands of years—around the world [1–5]. To this day, almost all school education programs throughout the world emphasize the importance of “early to bed and early to rise” to students, especially for children [6–8].

Indeed, previous studies on adolescents have shown that late bedtimes and late wake-up times are associated with poorer diet quality, unfavorable activities, and weight status profile, and these effects are independent of sleep duration, age, sex, household income, and geographical remoteness [9, 10]. However, studies of adult patients and elderly people have shown that being “early to bed and early to rise” is not associated with mortality, income, or educational attainment [11, 12]. With regards to the older adults, it is necessary to examine whether being “early to bed and early to rise” reduces physician visits, translating into lower health care use—a goal sought by policy makers, individuals, families, and societies. To our knowledge, the association between sleep–wake habits and the use of health care services has never been rigorously examined.

Although some proverbs are not fully supported by empirical evidence, we cannot deny all of the practical experience and wisdom of our predecessors [1]. After all, the theory of modern medicine is derived from the practice of our ancestors and builds on their work, therefore serving as a link between the past and the future. Therefore, in-depth research is necessary to eliminate the false and retain the true, separating out what is useful and essential among the aphorisms. In China, an aphorism closely related to “Early to bed and early to rise…” is “Do not seek an immortality prescription; look for a sleep prescription.” If these aphorisms hold true, sleep prescription could potentially improve health outcomes and thus reduce individual and national health care spending.

We used data from the Dongfeng-Tongji (DFTJ) cohort study to examine the association of sleep–wake habits with the use of health care services (physician visits and hospitalization). We aimed to provide more in-depth, precise evidence to test whether “Early to bed and early to rise…” are associated with better health of middle-aged and older adults.

## RESULTS

The maximum age of the participants was 94 years, the youngest age was 37 years, and the average age was 64.7 ± 8.6 years. Table 1 presents the characteristics of participants by sleep–wake habits. Those who were “early to bed” (including 43.0% Early-bed/Early-rise (EE) and 5.7% Early-bed/Late-rise (EL)) and “late to bed” (including 39.5% Late-bed/Early-rise (LE) and 11.8% Late-bed/Late-rise (LL)) each made up roughly half of the participants. The demographic characteristics and lifestyles of participants in the other three groups were significantly different from the EE group, except for sex and drinking status for the EL group (*P* =0.481 and 0.109, respectively). The average age of the participants by sleep–wake habits was 66.5 ± 8.2 years for EE, 65.2 ± 10.1 years for EL, 63.5 ± 8.1 years for LE, and 61.5 ± 8.9 years for LL. Compared with EE participants, the “late to bed” (including LE and LL) group included more women, highly educated people, and passive smokers, and fewer people with self-reported chronic diseases, former drinkers, and former smokers (all *P*<0.001). Exercise habits were more frequently observed among participants who were “early to rise” (88.6% for EE and 90.0% for LE) than among those who were “late to rise” (76.2% for EL and 84.5% for LL) (all *P*<0.001).

**Table 1:** Characteristics of participants according to sleep-wake habits.

**Characteristics of participants**	**Sleep–wake Habits**									
	**EE**		**EL**			**LE**			**LL**	
	**%[Ref]**		**%**	***P* value**		**%**	***P* value**		**%**	***P* value**
Total	43.0		5.7			39.5			11.8	
Demographic characteristics										
Age(Mean ± SD)	66.5±8.2		65.2±10.1		63.5±8.1		61.5±8.9
<60	19.6		30.3	<0.001		31.9	<0.001		43.2	<0.001
60-69	48.0		38.2			47.1			39.5	
≥70	32.5		31.5			21.0			17.3	
Sex										
Male	48.9		48.1	0.481		41.1	<0.001		40.3	<0.001
Female	51.1		51.9			58.9			59.7	
Education attainment										
Primary school and below	30.6		26.7	<0.001		16.5	<0.001		11.8	<0.001
Middle school	38.3		36.6			36.1			34.3	
High school	22.8		27.6			32.8			39.2	
College and above	8.3		9.1			14.6			14.7	
Marital status										
Married	86.7		82.8	<0.001		88.5	<0.001		88.1	0.017
Others	13.3		17.2			11.5			11.9	
Health-related characteristics										
Number of chronic diseases										
0	40.3		40.5	0.003		47.3	<0.001		50.5	<0.001
1	32.0		28.8			31.0			28.9	
≥2	27.7		30.7			21.7			20.6	
Smoking status										
Never smoker	70.8		64.8	<0.001		73.7	<0.001		70.5	<0.001
Current smoker	15.6		21.2			15.2			19.7	
Former smoker	13.6		14.1			11.1			9.7	
Passive smoking										
No	73.6		68.4	<0.001		67.2	<0.001		62.5	<0.001
Yes	26.4		31.6			32.8			37.5	
Drinking status										
Never drinker	69.9		69.1	0.109		71.0	<0.001		69.7	<0.001
Current drinker	23.5		23.0			23.6			25.6	
Former drinker	6.7		7.9			5.4			4.7	
Exercise										
No	11.4		23.8	<0.001		10.0	<0.001		15.5	<0.001
Yes	88.6		76.2			90.0			84.5	

EE: Early-bed/Early-rise; EL: Early-bed/Late-rise; LE: Late-bed/ Early-rise; LL: Late-bed/Late-rise.

Table 2 presents the use of health care services by sleep–wake habits. In total, 60.8% of the participants reported to visit doctors more than once, and the corresponding proportions of the four groups were 59.9%, 61.7%, 61.3%, and 62.1%, respectively (*P* = 0.020). There were 36.4% of the participants have been hospitalized in the past year, and the hospitalization rates for the four groups were 38.4%, 40.9%, 34.3%, and 33.4%, respectively (*P* < 0.001). In the full sample, compared with EE participants, LL participants were more likely to report physician visits (*P* = 0.011); the results of the gender- and age-stratified analyses were highly similar (except for women, with *P* = 0.060). In the full sample, LL participants were less likely than EE participants to report hospitalization (*P* < 0.001); the results of the gender-stratified analysis were similar, but the results of the age-stratified analysis differed. For participants who were younger than 60 years, there were no significant differences among the four groups. For participants aged 60–69 years, differences in hospitalization by sleep–wake habits were similar to those in the total sample. For participants aged ≥ 70 years, the difference in hospitalization between EE and LL was not statistically significant (*P* = 0.103).

**Table 2:** Use of health care services according to sleep-wake habits.

**Use of health care services**	**Sleep–wake Habits**									
	**EE**		**EL**			**LE**			**LL**	
	**%[Ref]**		**%**	***P* value**		**%**	***P* value**		**%**	***P* value**
Total										
Physician visits										
0	27.2		26.2	0.326		26.3	0.093		25.4	0.011
1	12.9		12.1			12.4			12.6	
2-3	23.8		23.5			24.7			26.6	
≥4	36.2		38.1			36.6			35.4	
Hospitalization										
0	61.6		59.1	0.002		65.6	<0.001		66.6	<0.001
1	22.7		22.2			22.3			21.9	
≥2	15.8		18.7			12.1			11.6	
Male										
Physician visits										
0	28.4		26.6	0.541		27.0	0.051		26.8	0.047
1	13.0		12.4			12.5			11.9	
2-3	22.4		23.5			24.2			25.4	
≥4	36.1		37.5			36.3			35.9	
Hospitalization										
0	61.0		57.5	0.043		64.9	<0.001		66.1	<0.001
1	22.7		23.5			22.4			20.8	
≥2	16.2		19.1			12.7			13.1	
Female										
Physician visits										
0	26.0		25.8	0.391		25.8	0.856		24.4	0.060
1	12.8		11.9			12.4			13.0	
2-3	25.0		23.6			25.0			27.5	
≥4	36.2		38.7			36.7			35.1	
Hospitalization										
0	62.1		60.5	0.028		66.2	<0.001		66.8	<0.001
1	22.6		21.1			22.1			22.6	
≥2	15.3		18.4			11.7			10.6	
Age<60 years old										
Physician visits										
0	35.1		34.9	0.180		31.5	<0.001		29.3	<0.001
1	14.9		12.7			14.2			14.7	
2-3	25.7		24.6			26.4			27.4	
≥4	24.3		27.9			27.8			28.6	
Hospitalization										
0	72.1		70.8	0.396		72.6	0.710		72.1	0.344
1	18.7		18.2			18.8			19.8	
≥2	9.2		10.9			8.6			8.1	
Age=60-69 years old										
Physician visits										
0	28.2		25.8	0.149		26.1	<0.001		25.2	<0.001
1	13.7		13.7			12.3			11.1	
2-3	23.5		23.3			24.3			25.9	
≥4	34.6		37.1			37.3			37.8	
Hospitalization										
0	63.7		59.8	0.010		65.8	<0.001		66.8	0.030
1	22.4		22.3			23.0			21.3	
≥2	13.9		17.8			11.2			11.9	
Age ≥70 years old										
Physician visits										
0	21.0		18.3	0.282		18.8	0.070		16.0	0.011
1	10.4		9.8			10.1			10.6	
2-3	23.0		22.7			23.0			26.3	
≥4	45.7		49.2			48.1			47.1	
Hospitalization										
0	52.2		46.8	0.009		54.9	0.004		52.1	0.103
1	25.4		25.9			25.8			28.3	
≥2	22.4		27.3			19.3			19.5	

EE: Early-bed/Early-rise; EL: Early-bed/Late-rise; LE: Late-bed/ Early-rise; LL: Late-bed/Late-rise.

**Figure 1:**
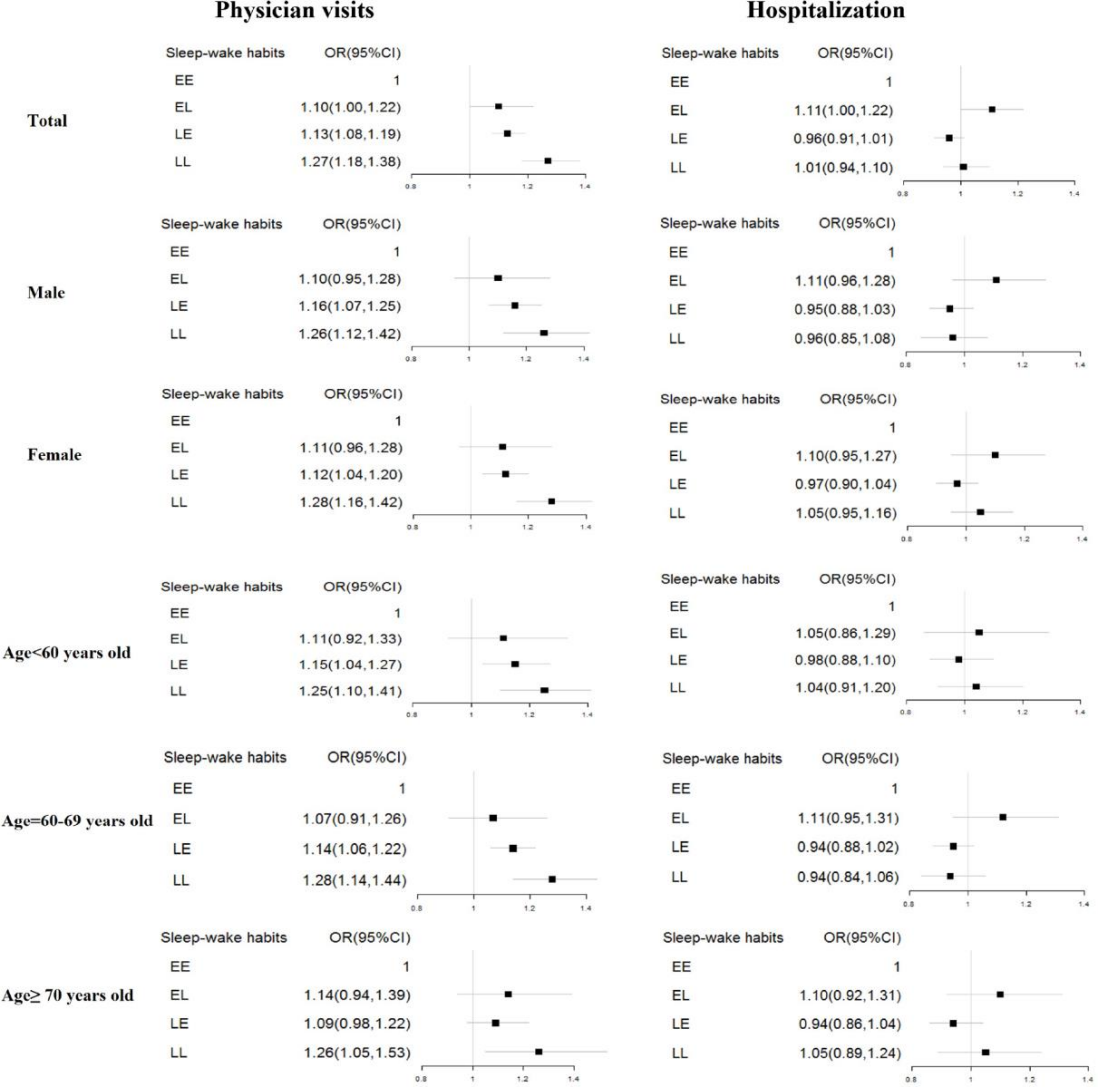
**The Association between sleep–wake habits and the use of health care services.** EE: Early-bed/Early-rise; EL: Early-bed/Late-rise; LE: Late-bed/ Early-rise; LL: Late-bed/Late-rise. The models for all participants adjusted for age, sex, educational attainment, and marital status, number of chronic diseases, smoking, passive smoking, drinking, and exercise. The stratified analysis adjusted for all the covariates except for the stratified variable.

To explore the associations between sleep-wake habits with the number of physician visits and hospitalization, we conducted multivariable logistic regression analyses by adjusting potential confounders (i.e., age, sex, educational attainment, marital status, number of chronic diseases, smoking, passive smoking, drinking, and exercise) (Figure 1). In the unadjusted analyses, EL, LE, LL participants were 1.08, 1.06, 1.09 times more likely to have a higher number of physician visits than EE participants though the effect for the EL group was not statistically significant (Supplementary Table 1). The odd ratio of hospitalization for EL participants was 11% (95%CI: 1.01–1.22) higher than the EE group. However, we found no such association for the LE and LL groups (LE: OR = 0.84, 95% CI :0.80–0.88; LL: OR = 0.81, 95% CI: 0.75–0.87). After adjusted for all the covariates, LE and LL participants were more likely to report higher number of physician visits than EE participants (OR = 1.13, 95% CI: 1.08–1.19 and OR = 1.27, 95% CI: 1.18–1.38, respectively); the results of the gender- and age-stratified analyses were highly similar (Figure 1). Compared with EE participants, hospitalization did not differ significantly for the other three categories of sleep–wake habits (EL: OR = 1.11, 95% CI: 1.00–1.22; LE: OR = 0.96, 95% CI :0.91–1.01; LL: OR = 1.01, 95% CI: 0.94–1.10); the results of the gender- and age-stratified analyses were highly similar.

## DISCUSSION

To our knowledge, this investigation was the first large-scale empirical study to examine the association between sleep–wake habits and physician visits. The results showed that, compared with EE, those who were late to bed, whether LL or LE, reported higher numbers of physician visits but not more frequent report hospitalization.

The lower number of physician visits by EE participants may result from the health effects of circadian rhythms, endogenous rhythms with a periodicity of approximately 24 hours [13–15]. Recent evidence strongly points to the pervasive influence of circadian timing on nearly all physiologic functions, including brain function, hormonal changes, mood disturbances, the endocrine system, and the metabolic process [16–19]. EE is considered the ideal sleep timing, in line with physiological circadian rhythms [20–22]. However, this ideal sleep timing has encountered serious challenges in the past few decades. With the rapid development of the Internet, increasing numbers of people stay up late taking part in activities such as chatting online, playing games, and watching videos [23, 24]. By 2013 when this present study was conducted, 71% of the Chinese population owns a mobile phone and social media has become embedded in ordinary people’s life [25]. Social media platforms such as WeChat has become a daily communication tool for most urban Chinese people (93%) including older adults [26]. Although the use of information and communicative technology (ICT) was found to enhance the psychological well-being of older adults, excessive use of ICT and social media can challenge the ideal sleep timing [27].

The difference in the impact of EE on physician visits and hospitalization reveals the applicable conditions of the proverb in health care services, similar to the indications for a medication or surgical procedure. The vast majority of aphorisms, including “Early to bed and early to rise makes a man healthy,” were generated in the context of crude medical practice. These experiences were not derived from dealing with serious illnesses or advanced stages of disease in clinical practice, but rather from the practice of dealing with minor illnesses or the early stages of disease or maintaining a condition of well-being. Therefore, the indication of “Early to bed and early to rise makes a man healthy” would be for pre-clinical practice with the minor illnesses or in the early stages of disease, to make the aphorism more precise for health care services.

Although the effect of EE is weak, it may nip the evil in the bud, and there may also be potential to reduce individual and national health care spending. If the observed association between sleep–wake habits and reduced physician visits is causal, the promotion of EE could, at least in theory, help to contain the costs associated with physician visits. In a rough and conservative calculation, based on Chinese data for the approximate average health care spending on physician visits for adults of $23 and annual physician visits in 2016 of 7.7 billion [28], we estimate that the savings in health care spending associated with a rise in EE would be $17 billion per year, with a 30% reduction in physician visits for LL and a 15% reduction in physician visits for LE. Given the approximate average travel expenses ($2) and the cost of lost working time ($15/day) for patients and their accompanying family members, the savings in indirect spending would be $136.14 billion per year. The total cost savings is about $153.14 billion per year.

Several potential limitations of the present study should be noted. First, our study sample consisted of middle-aged and elderly people, which is not representative of the general population. However, the consistency of the results from the age-stratified analysis reveals that the effects of sleep–wake habits on physician visits may not vary by age. Second, although we account in our analyses for a variety of health-related characteristics, we cannot dismiss the possibility of residual confounding. EE participants were, in fact, measurably different from others and would be expected to differ on other, unmeasured factors. Specifically, EE individuals may be more health-conscious and hold more industrious values, which could entirely explain the associations we observed. Third, because of the cross-sectional study design, we are unable to assess cause and effect in the observed relationships between EE and the successful avoidance of physician visits. However, we anticipate that causality is likely, given the universality of circadian rhythms for both men and women and in the past, present, and future [14, 29, 30].

The practice of medicine has developed greatly in the 21^st^ century. However, the health problems that human beings encounter are still complex and severe, and societies remain overburdened with the disease. We believe that it is very necessary to examine what we can learn from the past. Evidence from the present study shows that compared with EE, LL would increase about 30% physician visits. Additionally, our investigation allows us to carry forward the wisdom of our predecessors involved in the aphorism through revealing that the indication of “Early to bed and early to rise makes a man healthy” may be mainly in pre-clinical practice. This makes the aphorism more precise for health care services. Faced with an increasing number of young people staying up late and increasing health care spending in modern society, follow-up research on these topics would be of particular value to young people as the workforce of today and the patients of tomorrow.

## CONCLUSION

This study found that middle-aged and elderly people who stayed up late and got up late are more likely to visit the doctors than those who went to bed early and got up early. However, it is also possible that early to bed and early to rise individuals may be more health-conscious, which could explain the associations observed. Nevertheless, this is the first large-scale empirical study that provided rigorous evidence to support the association between sleep–wake habits and the use of health care services for middle-aged and older adults in China. Limited to the cross-sectional design, we cannot interpret such association as causality. Moreover, we cannot eliminate bias due to unmeasured confounders. Further prospective cohort studies are needed to validate our findings through the rigorous design and comprehensive consideration of potential confounders.

## MATERIALS AND METHODS

### Data

We obtained data from the DFTJ cohort study, a prospective cohort study in Hubei Province, China. The details of this cohort have been described in a previous report [31]. Briefly, 27,009 retired employees from the Dongfeng Motor Corporation (DMC) were recruited and completed baseline questionnaires from September 2008 to June 2010. All participants were covered by the health-care service system of DMC and urban employee-based basic medical insurance, which provides them with fair access to health care services and financial protection. In-person interviews were conducted with the study participants. The participants repeated the questionnaire interviews at follow-up investigations every 5 years. The content of this questionnaire included details about sociodemographic characteristics, lifestyle, occupational history, environmental exposure, and medical history. The first follow-up investigation was completed in 2013. In the present study, we analyzed data from all retired employees who participated in the 2013 investigation. We excluded 1694 (4.42%) individuals because of incomplete information on sleep and health care use. The final valid sample comprised of 36,601 (95.58%) responses.

The study was approved by the Medical Ethics Committee of the School of Public Health, Tongji Medical College, Huazhong University of Science and Technology and DMC affiliated Dongfeng General Hospital, and was subsequently carried out in accordance with the approved guidelines. All participants provided written informed consent.

### Measures

To test the impact of sleep–wake habits on the use of health care services directly, bedtimes, rather than sleep times/durations, were used [10]. As a measure of usual bedtime, participants reported the time that they usually went to bed at night and the time that they usually woke up in the last 6 months. Based upon China's social traditions and time lag, we considered participants who reported a usual bedtime earlier than 10:00 p.m. to be early to bed, and those who reported a usual waking time earlier than 7:00 a.m. to be early to rise. We defined a bedtime as 10:00 p.m. or later to be late to bed, a waking time as 7:00 a.m. or later to be late to rise. We categorized the study participants into four groups based on sleep timing behaviors: early to bed and early to rise (EE), early to bed and late to rise (EL), late to bed and early to rise (LE), and late to bed and late to rise (LL).

We operationalized our primary outcome as participants’ numbers of physician visits and hospitalizations in the past year, reflecting whether the participant felt sick and whether the participant was seriously ill, respectively. These measures are more objective, compared with perceived health, and more valuable than mortality to potentially reduce health care spending. We examined self-reported use of health care services with two questions: “How many times did you see a doctor because of your health at any location in the past year?” and “How many times have you been hospitalized because of your health at any location in the past year?”. The participants were asked to select from number 0 to 10 or more to reflect the times they visited the doctors in the past year and we grouped them into four categories: none, once, two to three times, four times or more. The participants were asked to select from number 0 to 6 or more to reflect the times they were hospitalized in the past year. Based upon the distributions of the use of health care services in the cohort population, we consolidated the number of hospitalizations into three categories: none, once, twice or more.

We identified covariates to address potential confounding by sociodemographic characteristics (age, sex, educational attainment, and marital status) and health-related characteristics (number of chronic diseases, smoking, passive smoking, drinking, and exercise). Chronic diseases included self-reported hypertension, diabetes, coronary heart disease, myocardial infarction, stroke, cancer, chronic hepatitis, and nephritis [31].

### Statistical analysis

Continuous variables are presented as the mean and standard deviation, and categorical variables are shown as percentages. For crude comparisons, we used chi-square tests for categorical variables and analysis of variance for continuous variables. Logistic regression models were conducted to determine the association between sleep patterns and the use of health care services (coding physician visits as 0 or 1 visit vs. ≥2 visits, and hospitalization as no visits vs. any visits). Odds ratios (ORs) and 95% confidence intervals (CIs) were presented for the unadjusted and adjusted regression models.

We conducted a gender-stratified analysis to examine whether there was a difference in the association between sleep-wake habits and the use of health care services for males and females. Additionally, we also performed an age-stratified analysis (defined as <60, 60-69 and ≥70 years old), considering the large age range (37–97 years old) of the participants and the effects of age on sleep, disease, and the use of health care services [32–34]. All analyses were performed using IBM SPSS Statistics, version 22. Statistical tests were two-tailed, and differences were considered significant with *P* <0.05.

## Supplementary Material

Supplementary Table 1
