# Supplementary material for: Association between sleep–wake habits and use of health care services of middle-aged and elderly adults in China
Source: Aging (Albany NY). 2020 Feb 24;12(4):3926–35. doi: 10.18632/aging.102860 (PMC7066913; doi:10.18632/aging.102860)
Supplement: Supplementary Table 1 [file aging-12-102860-s001..pdf]

SUPPLEMENTARY TABLE

Supplementary Table 1. The association between sleep–wake habits and the use of health care services (crude models).

Sleep–Wake Habits	Physician Visits OR(95%CI)	Hospitalization OR(95%CI)
**Total**		
EE	1.00(reference)	1.00(reference)
EL	1.08(0.98,1.18)	1.11(1.01,1.22)
LE	1.06(1.01,1.11)	0.84(0.80,0.88)
LL	1.09(1.02,1.17)	0.81(0.75,0.87)
**Male**		
EE	1.00(reference)	1.00(reference)
EL	1.11(0.97,1.27)	1.16(1.01,1.32)
LE	1.08(1.01,1.16)	0.85(0.79,0.91)
LL	1.12(1.01,1.25)	0.80(0.72,0.89)
**Female**		
EE	1.00(reference)	1.00(reference)
EL	1.05(0.92,1.19)	1.07(0.94,1.22)
LE	1.02(0.96,1.09)	0.84(0.79,0.89)
LL	1.06(0.97,1.16)	0.81(0.74,0.89)
**Age < 60 years**		
EE	1.00(reference)	1.00(reference)
EL	1.10(0.93,1.31)	1.06(0.88,1.28)
LE	1.18(1.08,1.30)	0.98(0.88,1.08)
LL	1.27(1.14,1.43)	1.00(0.88,1.14)
**Age=60-69 years**		
EE	1.00(reference)	1.00(reference)
EL	1.11(0.95,1.28)	1.18(1.01,1.37)
LE	1.16(1.08,1.24)	0.91(0.85,0.98)
LL	1.27(1.14,1.41)	0.87(0.78,0.97)
**Age ≥ 70 years**		
EE	1.00(reference)	1.00(reference)
EL	1.17(0.98,1.40)	1.24(1.06,1.46)
LE	1.13(1.02,1.24)	0.90(0.82,0.98)
LL	1.26(1.06,1.50)	1.00(0.86,1.17)

EE: Early-bed/Early-rise; EL: Early-bed/Late-rise; LE: Late-bed/ Early-rise; LL: Late-bed/Late-rise.
